# Mitofusin 1-Mediated Redistribution of Mitochondrial Antiviral Signaling Protein Promotes Type 1 Interferon Response in Human Cytomegalovirus Infection

**DOI:** 10.1128/spectrum.04615-22

**Published:** 2023-03-20

**Authors:** Kaizhao Huang, Shunjie Pei, Yi Sun, Xi Xu, Yangyang Fang, Meimei Lai, Guangxin Xiang, Feng Xu, Xiaoqun Zheng

**Affiliations:** a Second Affiliated Hospital and Yuying Children’s Hospital, Wenzhou Medical University, Wenzhou, Zhejiang, China; b School of Laboratory Medicine and Life Sciences, Wenzhou Medical University, Wenzhou, Zhejiang, China; c Key Laboratory of Laboratory Medicine, Ministry of Education, Wenzhou, Zhejiang, China; d Department of Clinical Laboratory, Wenzhou People’s Hospital, Third Clinical Institute affiliated with Wenzhou Medical University, Wenzhou, China; University of Nevada Reno

**Keywords:** human cytomegalovirus, mitofusin 1, mitochondrial antiviral signal protein, type I interferon

## Abstract

One of the most potent anti-human cytomegalovirus (HCMV) immune mechanisms possessed by host cells is type I interferon (IFN1), which induces the expression of IFN-stimulated genes (ISGs). During this process, mitochondria play an important role in the IFN1 response, and mitofusin 1 (MFN1) is a key regulator of mitochondrial fusion located on the outer mitochondrial membrane. However, the underlying mechanism of MFN1’s promotion of IFN1 during HCMV infection still remains unknown. In this study, HCMV infection promoted IFN1 production and enhanced ISG expression. Meanwhile, it promoted the increase of mitochondrial fusion in THP-1 cells and peripheral blood mononuclear cells (PBMCs), especially the expression of MFN1. Phosphorylation of tank binding kinase 1 (p-TBK1), interferon regulatory factor 3 (p-IRF3), and ISGs was significantly decreased in MFN1 or mitochondrial antiviral signaling protein (MAVS)-knockdown THP-1 cells, and MFN1 was constitutively associated with MAVS, positively regulated mitochondrial fusion, and IFN1 production. Knockdown of MFN1 inhibited the MAVS redistribution without affecting the MAVS expression, whereas the HCMV-induced IFN1 production decreased. Conversely, leflunomide could induce the expression of MFN1, thereby producing IFN1 and stimulating the expression of ISG in leflunomide-treated THP-1 cells. These observations reveal that HCMV infection leads to MFN1-mediated redistribution of MAVS and then induces an antiviral response of IFN1 and that the MFN-agonist leflunomide promotes IFN1 responses and may serve as a potential anti-HCMV therapy.

**IMPORTANCE** Human cytomegalovirus (HCMV) infection is ubiquitous and is often asymptomatic in healthy individuals, but it can cause great damage to newborns, AIDS patients, and other immune deficiency patients. In this study, we found that HCMV infection caused mitochondrial fusion, and expression of mitofusin 1 (MFN1), which is a protein associated with mitochondrial antiviral signaling protein (MAVS), positively regulates mitochondrial fusion and HCMV-induced IFN1 response. Knockdown of MFN1 or MAVS can inhibit the HCMV-induced IFN1 production. What is more, confocal laser-scanning microscope showed that knockdown of MFN1 inhibits the HCMV-induced redistribution of MAVS. Conversely, MFN1 agonist leflunomide could induce IFN1 production. In conclusion, we provide new insight into the relationship between MFN1 and IFN1 during HCMV infection and show that MFN1 may serve as a potential strategy against HCMV infection.

## INTRODUCTION

Human cytomegalovirus (HCMV) is a highly prevalent opportunistic and restricted species specificity pathogen whose main hosts are myeloid-derived cells (1, 2). During infection, the innate immunity will be induced through the production of type I interferon (IFN1) (3). IFN1 bound to the receptors on the cell membrane to activate the cell membrane adenylate cyclase, promoted the increase of adenylate cyclase, and activated a variety of interferon-stimulating genes (ISGs) to play an antiviral effect (4). IFN1 production in HCMV infection is triggered by cytoplasmic sensors such as cyclic GMP-AMP synthase (cGAS) and retinoic acid inducible gene I (RIG-I)-like receptor (RLR), which specifically sense viral DNA or RNA and induce antiviral signaling (4, 5). Their signals are relayed through physical interaction with some interferon gene-stimulating proteins, such as stimulator of interferon genes (STING) or mitochondrial antiviral signaling protein (MAVS) (6, 7). Previous studies showed that the MAVS was also activated by intermediate RNA products of some DNA viruses, such as herpes simplex virus 1 (HSV-1) and Epstein-Barr virus (8, 9), but few works revealed that MAVS plays an important role in HCMV-induced IFN1 production.

Mitochondria undergo dynamic fission and fusion in order to drive cellular metabolism (10), which is regulated by a variety of related proteins, including mitofusin 1 (MFN1), optic atrophy 1 (OPA1), and others (11). MAVS is located on the outer mitochondrial membrane, and the location of MAVS is essential to its function (12), which implies that mitochondrial dynamics may play an important role in innate immunity. As viral infection could cause mitochondrial fusion fission imbalance (13), and MFN1 is a GTPase protein located on the outer membrane of mitochondria, its expression is increased in HCMV infection, which may be involved in the localization of MAVS. Thus, we hope to investigate the function of MFN1 protein during HCMV infection and its association with IFN1 production.

Although previous studies have shown that IFN1 plays an important role in the inhibition of cytomegalovirus infection in mouse models (14), because of the major differences between HCMV and murine cytomegalovirus, in particular their interaction with the immune system, HCMV infection cannot be established in mice (15). In the present study, human monocytic macrophage THP-1 was used as cell model for HCMV infection and changes in interferon signal pathway and mitochondrial morphology, MFN1, and MAVS were detected. In addition, we detected the expression of MFN1 of monocytes in patients with HCMV infection. We aimed to illustrate the role of MFN1 in IFN response in HCMV infection.

## RESULTS

### HCMV infection promoted IFN1 production and induced the expression of MFN1 in THP-1 cells and human monocytes.

**FIG 1:**
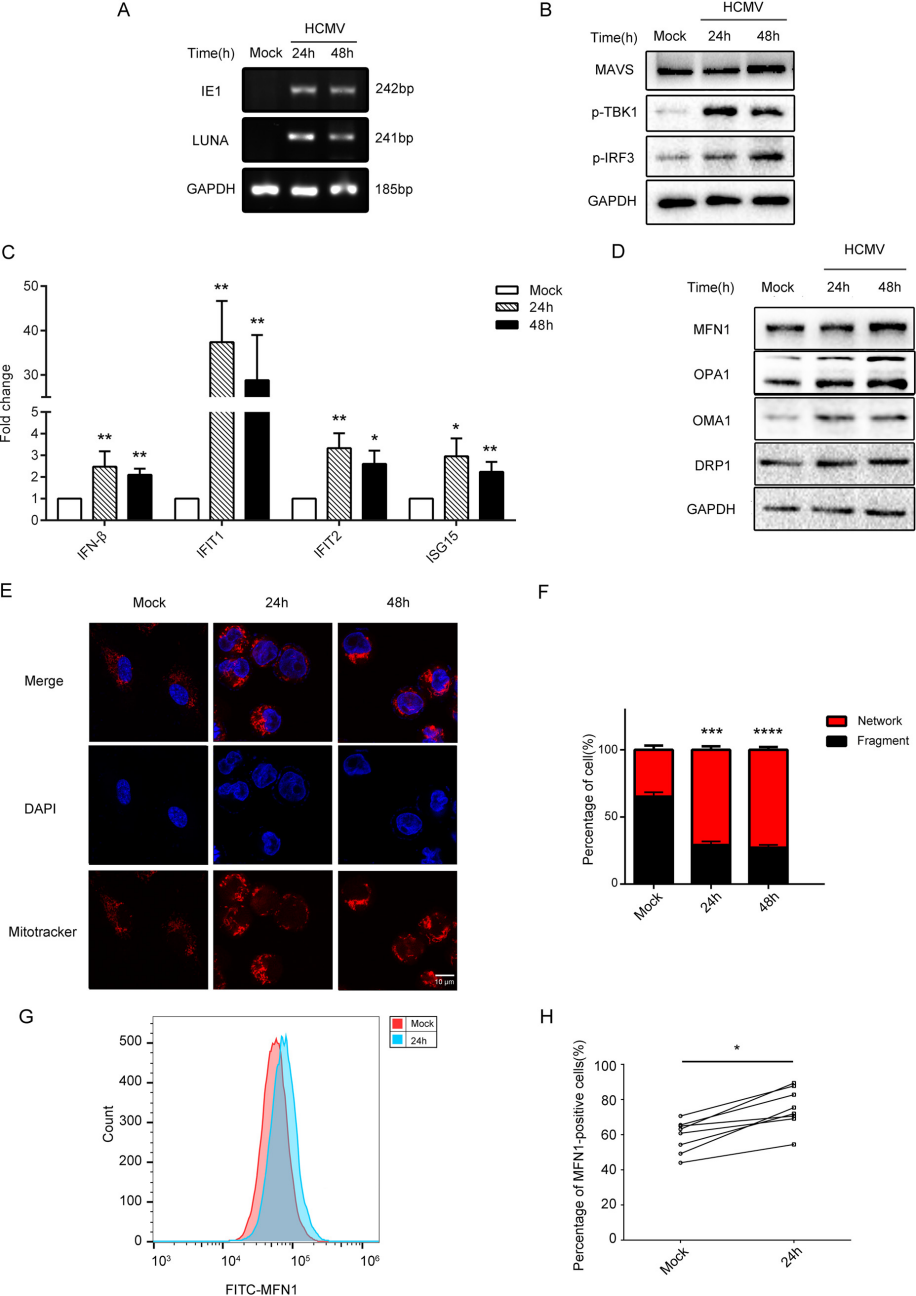
HCMV infection caused the imbalance of mitochondrial fusion division and induced the expression of MFN1 in THP-1 cells and human monocytes. (A) The levels of HCMV transcripts (*IE1* and *LUNA* mRNA) analyzed by RT-PCR. (B) Western blot of MAVS, p-TBK1, p-IRF3 in lysates from control, and THP-1 cells infected with HCMV (MOI = 5) for 24 or 48 h. GAPDH was used as a loading control. (C) The levels of HCMV transcripts (*IFN-β*, *IFIT1*, *IFIT2* and *ISG15* mRNA) analyzed by RT-PCR. (D) Western blot of MFN1, OPA1, OMA1, and DRP1 in lysates from control and THP-1 cells infected with HCMV (MOI = 5) for 24 or 48 h. GAPDH was used as a loading control. (E) THP-1 cells were infected with HCMV (MOI = 5) for 24 or 48 h, stained, and subjected to confocal microscopy. Uninfected cells serve as a mock control. (F) Mitochondrial perimeter measurements were obtained from multiple independent images and graphed as a percentage of the total number of mitochondria counted for each sample. (G, H) Flow cytometry analysis of the intracellular MFN1 protein expression from donors’ peripheral blood mononuclear cells infected with HCMV (MOI = 5) for 24 h. The data are shown as the means ± standard error of the mean (SEM) of three independent experiments. DAPI, 4′,6-diamidino-2-phenylindole; FITC, fluorescein isothiocyanate; GAPDH, glyceraldehyde-3-phosphate dehydrogenase; HCMV, human cytomegalovirus; IE, immediate early gene; IFIT, interferon-induced protein with tetratricopeptide repeat; IFN, interferon; ISG, IFN-stimulated gene; LUNA, latency unique nuclear antigen; MAVS, mitochondrial antiviral signaling protein; NS, not significant; *P > *0.05; *, *P < *0.05; **, *P < *0.01; ***, *P < *0.001.

In this study, the HCMV infection THP-1 cell model was constructed (Fig. 1A). Consistent with previous studies, the level of IE1 and LUNA mRNA increased after infection (16). In the HCMV-infected cell lysates, the MAVS, phosphorylation of TANK binding kinase 1 (p-TBK1) and phosphorylation of interferon regulatory factor 3 (p-IRF3) levels were increased (Fig. 1B; Fig. S2A). Meanwhile, the mRNA expression of interferon β (*IFN-β*), interferon-induced protein with tetratricopeptide repeats 1 (*IFIT1*), interferon-induced protein with tetratricopeptide repeats 2 (*IFIT2*), and ISG15 ubiquitin-like modifier (*ISG15*) were also significantly increased (Fig. 1C).

HCMV infection could induce increases of mitochondrial fusion and decreases of division (*P < *0.001) (Fig. 1E and F). Meanwhile, HCMV infection upregulated the expression levels of MFN1 and OPA1 and overlapped with the M-AAA protease 1 homolog (OMA1). However, the expression levels of dynamin-related protein 1 (DRP1) remained unchanged (Fig. 1D; Fig. S2B). This result is consistent with transcriptome GSE132048 data from the GEO database (17) (Fig. S1). Moreover, HCMV infection upregulated the expression levels of MFN1 in human monocytes (Fig. 1G and H; Fig. S3).

### Knockdown of MFN1 inhibited mitochondrion fusion and HCMV-induced IFN1 production.

**FIG 2:**
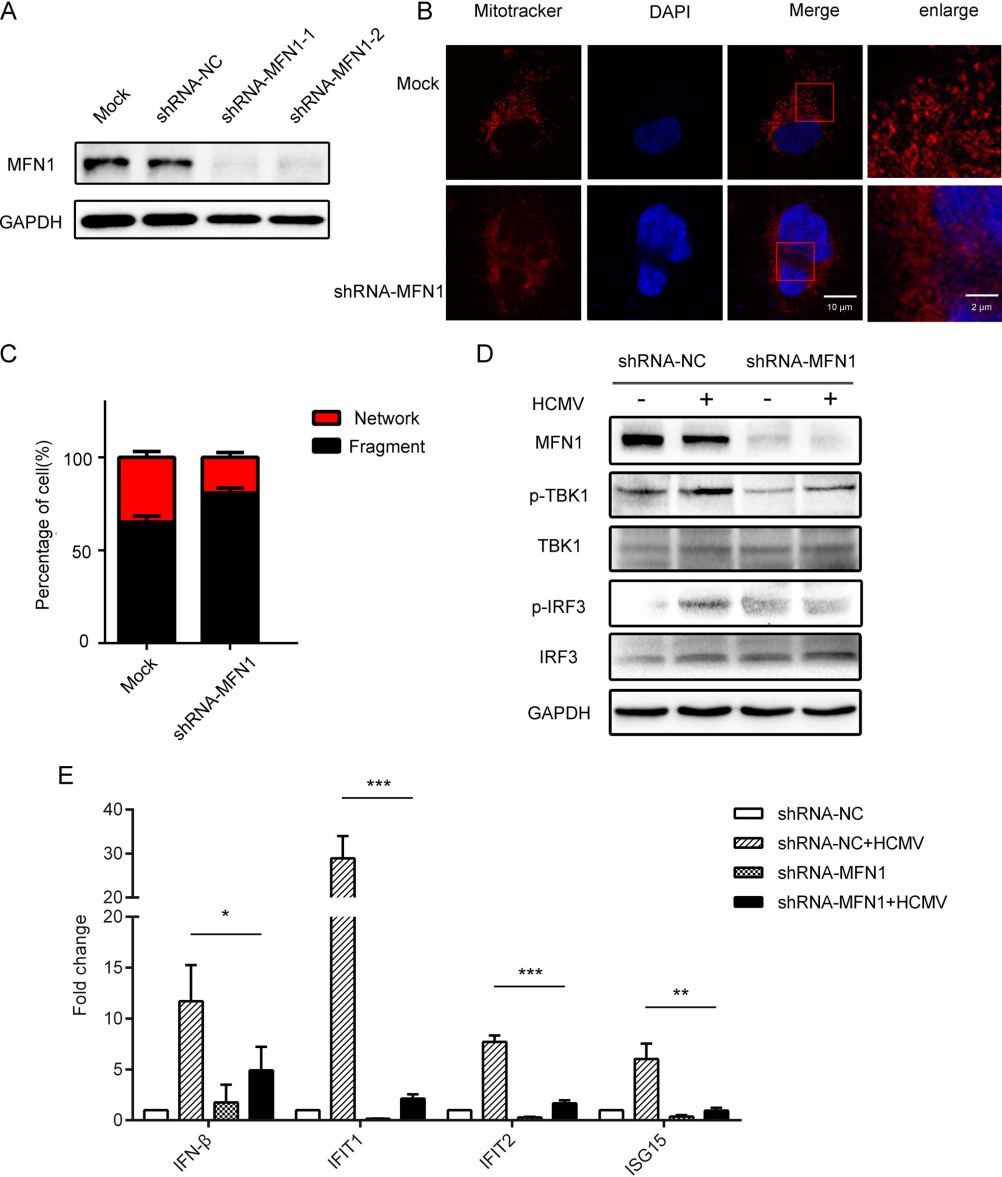
Knockdown of MFN1 inhibited mitochondrion fusion and HCMV-induced IFN1 production. (A) Western blot of MFN1 in lysates from control and MFN1-knockdown THP-1 cells. (B) MFN1-knockdown THP-1 cells and control shRNA-treated cells were stained and subjected to confocal microscopy. (C) Mitochondrial perimeter measurements were obtained from multiple independent images and graphed as a percentage of the total number of mitochondria counted for each sample. (D) Western blot of MFN1, p-TBK1, TBK1, p-IRF3, and IRF3 in lysates from control shRNA-treated and MFN1-knockdown THP-1 cells infected with HCMV (MOI = 5) for 48 h. GAPDH was used as a loading control. (E) The control shRNA-treated and MFN1-knockdown THP-1 cells were infected with HCMV (MOI = 5) for 48 h and analyzed for *IFN-β*, *IFIT1*, *IFIT2*, and *ISG15* mRNA expression relative to *GAPDH* mRNA by quantitative PCR. The data are shown as the means ± SEM of three independent experiments. NS, not significant; shRNA, short hairpin RNA; *P > *0.05; *, *P < *0.05; **, *P < *0.01; ***, *P < *0.001.

MFN1 is located on mitochondria (Fig. S2C), and confocal laser-scanning microscope shows increased mitochondrial fission in MFN1 knockdown cells (Fig. 2A and B). To confirm that mitochondrial alteration was needed for IFN1 production, we measured MFN1, p-TBK1, TBK1, p-IRF3, and IRF3 protein in MFN1-knockdown THP-1 cells. The results showed that the knockdown of MFN1 in THP-1 cells caused downregulation of IFN1 in the cytoplasm (Fig. 2C; Fig. S2D), and the mRNA expression of *IFN-β*, *IFIT1*, *IFIT2*, and *ISG15* was lower compared with the control group (Fig. 2D), suggesting that the MFN1 protein was involved in HCMV-induced IFN1 production.

### MFN1 altered the distribution of MAVS and promoted IFN1 production.

**FIG 3:**
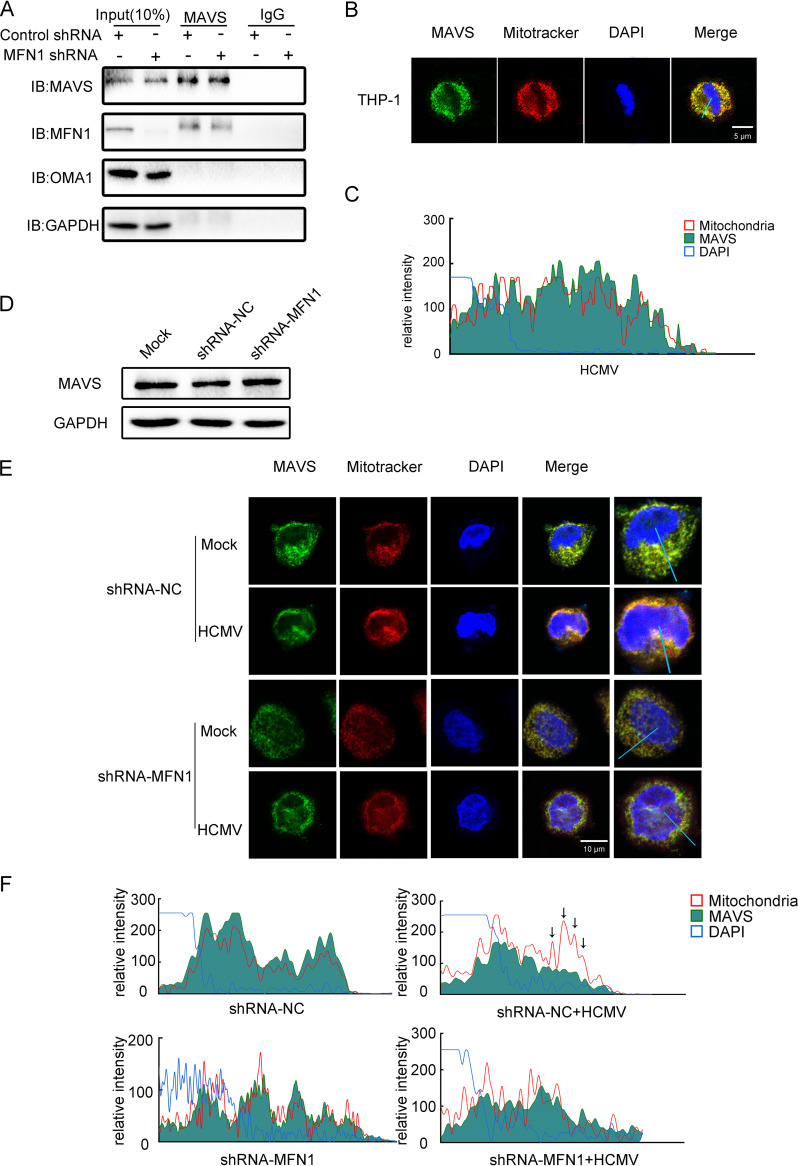
MFN1 altered the distribution of MAVS and promoted IFN1 production. (A) Coimmunoprecipitation analysis of MAVS and MFN1 in lysates from control shRNA-treated and MFN1-knockdown THP-1 cells. (B) THP-1 cells were stained with MitoTracker (mitochondria) and anti-MAVS antibody. The nuclei were visualized by staining with DAPI throughout this study. (C) The fluorescent image was quantified in the area indicated by blue line (right). (D) Western blot of MAVS in lysates from MFN1-knockdown THP-1 cells infected with HCMV (MOI = 5) for 48 h. GAPDH was used as a loading control. (E) The control shRNA-treated and MFN1-knockdown THP-1 cells were infected with HCMV (MOI = 5) for 48 h and stained with MitoTracker (mitochondria), anti-MAVS, and DAPI throughout this study. (F) The fluorescent image was quantified in the area indicated by blue line (right). IB, immunoblot.

Coimmunoprecipitation assay showed that MFN1 was constitutively associated with MAVS in the cells (Fig. 3A), and confocal microscopy showed that MAVS was localized on the outer membrane of mitochondria (Fig. 3B) (18). Interestingly, knockdown of MFN1 did not affect the expression of MAVS (Fig. 3C), implying that the altered localization of MAVS affected IFN1 production. Indeed, confocal microscopy revealed that redistribution of MAVS was observed, while control short hairpin RNA (shRNA)-treated cells were infected with HCMV, but not in MFN1-knockdown cells (Fig. 3D). Furthermore, two independent siRNA were used to knockdown MAVS expression (Fig. S2E), and the mRNA expression of *IFN-β*, *IFIT1*, *IFIT2*, and *ISG15* significantly decreased (Fig. S2F). Therefore, the above results strongly suggest that MFN1 was critical for the redistribution of MAVS and mediation of the IFN1 production.

### Mitochondrial fusion agonist potentiated the HCMV-induced IFN1 production.

**FIG 4:**
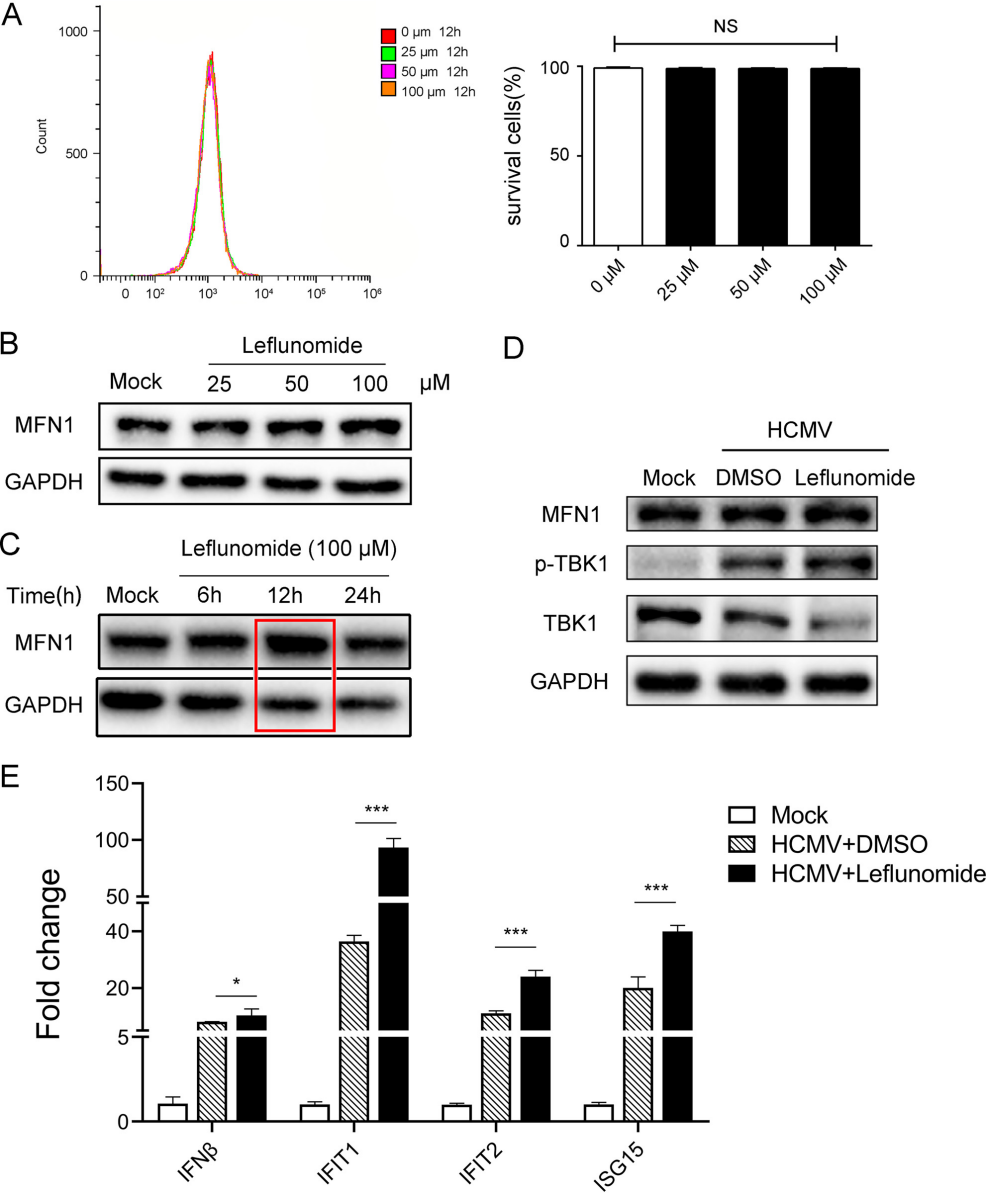
Mitochondrial fusion agonist potentiated the HCMV-induced IFN1 production. (A) In 7-aminoactinomycin D (7-AAD)-labeled cells, the cell survival rate was determined by flow cytometry. (B) THP-1 cells were pretreated indicated concentrations of leflunomide for 24 h before immunoblotting analysis. (C) THP-1 cells were pretreated leflunomide (100 μM) for the indicated times before immunoblotting analysis. (D) THP-1 cells were untreated or pretreated with leflunomide (100 μM) for 12 h and then infected with HCMV (MOI = 5) for the 48 h before immunoblotting analysis. (E) THP-1 cells were untreated or pretreated with leflunomide (100 μM) for 12 h; then infected with HCMV (MOI = 5) for the 48 h; and analyzed for *IFN-β*, *IFIT1*, *IFIT2*, and *ISG15* mRNA expression relative to GAPDH mRNA by quantitative PCR. DMSO, dimethyl sulfoxide.

The MFN1-specific agonist leflunomide does not affect the cell viability of THP-1 cells (Fig. 4A) and could induce MFN1/2-dependent mitochondrial fusion by inhibiting dihydroorotate dehydrogenase (DHODH) (19), thereby significantly increasing the expression of MFN1 and p-TBK1 and the mRNA expression of *IFN-β*, *IFIT1*, *IFIT2*, and *ISG15* in HCMV-infected THP-1 cells treated with leflunomide (Fig. 4B to E). This suggested that HCMV-induced IFN1 production was mediated by MFN1.

## DISCUSSION

In present study, we evaluated the transcription of HCMV genome in infected THP-1 cells over a 48-h course of infection. The levels of IE1 and LUNA mRNA were determined for viral gene transcription levels, which were continuously expressed throughout the infection process, indicating the successful construction of the HCMV infection model (16). HCMV infection could induce the host innate immune response, producing IFN1 and increasing mitochondrial fusion. During these processes, MFN1 positively regulates mitochondrial fusion and IFN1 production. Knockdown of MFN1 or MAVS inhibited the HCMV-induced IFN1 production and redistribution of MAVS. Conversely, MFN1-agonist leflunomide could induce IFN1 production.

Our results are similar to HSV-1, which is a herpesvirus that impaired the mitochondrial network through the viral protein UL12.5, leading to the fusion of mitochondria (20). The expression levels of mitochondrial fusion-related proteins including MFN1, OPA1, and OMA1 were increased in HCMV-infected THP-1 cells, as well as mitochondrial fusion after HCMV infection. Therefore, HCMV may also destroy mitochondria network through a certain viral protein, such as viral mitochondria-localized inhibitor of apoptosis (vMIA) protein, leading to mitochondrial fusion (21). We also found that MFN1 expression was higher in HCMV-infected human monocytes, indicating that mitochondrial fusion was important for HCMV replication and related to host immunity, which was in accordance with the previous report that mitochondrial fusion includes outer membrane fusion and inner membrane fusion, while the former was mediated by MFN1(22).

The phenomenon was observed in our study that knockdown of MAVS inhibited IFN1 production. As was reported, many interferons signaling pathway proteins, such as STING and MAVS, are located on the outer membrane of mitochondria (7, 18), which can regulate innate immune signal pathway (23, 24). Once viral infection occurs, MDA5 and RIG-I recognize viral RNA and transmit signals to MAVS, and MAVS aggregation can regulate the expression of IFN1 (6, 12, 25). In addition, RNA polymerase III recognized DNA in cytoplasm and stimulated innate immune responses through the RIG-I pathway (8, 9, 26). HCMV may thus activate the RIG-I pathway by transcribing RNA polymerase III. We hope such a regulatory mechanism can be elaborated in the future.

Consistent with previous reports (27), our results confirmed the importance of mitochondria in innate immunity. The MAVS protein was localized on the mitochondrial membrane and interacted with MFN1. HCMV infection regulated IFN1 expression through mitochondrial dynamics, whereas MFN1-knockdown THP cells induced mitochondrial fission and suppressed ISGs transcription and protein expression. In addition, the redistribution of MAVS was induced by HCMV infection, whereas the knockdown of MFN1 inhibited the redistribution of MAVS and disrupted the interferon signaling pathway. With redistribution of MAVS dependent on functional MFN1, the possible mechanism of HCMV affecting the upstream transcriptional expression of MFN1 might be through some encoded proteins (28), which caused the conformational change of MFN1 and then indirectly bound MAVS to cause redistribution. This process requires further proteomic studies to explore its functional mechanism.

Our results also showed that MFN1-specific agonist leflunomide enhanced IFN1 production. Leflunomide is also known as an anti-inflammatory drug used against rheumatoid arthritis, which increases mitochondrial elongation by depleting the pyrimidine pool and altering MFN1/2 protein levels through loss of pyrimidine synthesis (19, 29). In addition, with the consequence of MFN1 knockdown put into consideration, MFN1 might be a pharmacological target in the treatment of HCMV.

In summary, our study provides new insight into the relationship between MFN1 and IFN1 during HCMV infection, with regulating of the balance between mitochondrial dynamics and IFN1 proven to be critical. We also provide evidence that the functions of mitochondrial dynamics are not only restricted to regulation of the transmission of mitochondrial DNA (30) but also include the regulation of antiviral signals, thereby opening a new potential strategy against HCMV infection.

## MATERIALS AND METHODS

### Cells culture and virus infection.

THP-1 cells were cultured in RPMI 1640 medium (Life Technologies, Grand Island, NY) containing 10% fetal bovine serum (FBS, Life Technologies) and antibiotics (100 units/mL penicillin and 100 μg/mL streptomycin) at 37°C at an atmosphere of 5% CO_2_. HCMV Towne strain was purified by sedimentation through sucrose gradient, and virus titer is measured by median tissue culture infectious dose (TCID_50_) (31). THP-1 cell line was infected with HCMV Towne at a multiplicity of infection (MOI) of 5 and collected at the indicated time points.

### RNA isolation and PCR.

The total RNA was isolated from cells using TRIzol (ThermoFisher Scientific, Waltham, MA) according to the manufacturer’s protocol. After extraction, extracted RNA was reverse-transcribed using the reverse transcription (RT) kit (TaKaRa, Shiga, Japan), and real-time quantitative PCR was performed using Fast SYBR green Master Mix (TaKaRa, Shiga, Japan). The expression values of each replicate were normalized against GAPDH cDNA using the 2^−ΔΔCt^ method. The primers used for RT-PCR are shown in Table 1. Each biological sample was repeated three times independently.

**TABLE 1:** Primers used for mRNA RT-PCR amplification of HCMV mRNAs^*a*^

Primer name	Nucleotide sequence (5′ to 3′)
GAPDH forward	AAAATCAAGTGGGGCGATGCT
GAPDH reverse	GGGCAGAGATGATGACCCTTT
IFNβ forward	GCTTGGATTCCTACAAAGAAGCA
IFNβ reverse	ATAGATGGTCAATGCGGCGTC
IFIT1 forward	TCAGGTCAAGGATAGTCTGGAG
IFIT1 reverse	AGGTTGTGTATTCCCACACTGTA
IFIT2 forward	GGAGGGAGAAAACTCCTTGGA
IFIT2 reverse	GGCCAGTAGGTTGCACATTGT
ISG15 forward	ATGGGCTGGGACCTGACGG
ISG15 reverse	TTAGCTCCGCCCGCCAGGC

a GAPDH, glyceraldehyde-3-phosphate dehydrogenase; HCMV, human cytomegalovirus; IFIT, interferon-induced protein with tetratricopeptide repeat; IFN, interferon; ISG, IFN-stimulated gene; RT, reverse transcription.

A 1-μL volume of cDNA from each sample was used for PCR amplification of latency unique nuclear antigen (LUNA) in a master mix solution containing 2× TaqMaster Mix (Vazyme, Nanjing, China) and adding the specific LUNA primers (LUNA round 1) listed in Table 2. Amplification conditions were denaturation at 94°C for 3 min followed by 20 cycles of 94°C for 30 s, 51.8°C for 30 s, and 72°C for 30 s and a final extension at 72°C for 5 min. Nested PCR was performed on 1 μL of the LUNA RT-PCR product, using the LUNA internal primers (LUNA round 2) listed in Table 2. Amplification conditions were as follows: denaturation at 94°C for 3 min followed by 30 cycles of 94°C for 30 s, 54.2°C for 30 s, and 72°C for 30 s and a final extension at 72°C for 5 min. Amplification of immediate early gene 1 (IE1) and GAPDH used the same denaturation and final extension conditions, with 35 cycles of 95°C for 10 s, 60°C for 15 s, and 72°C for 30 s.

**TABLE 2:** Primers used for PCR amplification of HCMV mRNAs^*a*^

Primer name	Nucleotide sequence (5′ to 3′)
IE1 forward	CAAGAGAAAGATGGACCCTG
IE1 reverse	CGAGTTCTGCCAGGACATC
LUNA round 1 forward	ATGACCTCTCCTCCACACC
LUNA round 1 reverse	GACGCTATATTTAGGGCTTCC
LUNA round 2 forward	GAGCCTTGACGACTTGGTAC
LUNA round 2 reverse	GGAAAAACACGCGGGGGA
GAPDH forward	GAGTCAACGGATTTGGTCGT
GAPDH reverse	TTGATTTTGGAGGGATCTCG

a GAPDH, glyceraldehyde-3-phosphate dehydrogenase; LUNA, latency unique nuclear antigen.

### Coimmunoprecipitation and immunoblotting.

Cell lysates were incubated with primary antibodies overnight at 4°C, and then protein A-Agarose (Beyotime, Shanghai, China) was added to the samples for 3 h at 4°C. The beads were washed five times with phosphate-buffered saline (PBS). The proteins were eluted by boiling in 1× denaturing buffer (50 mM Tris-HCl, pH 6.8, 2% SDS, 5% β-mercaptoethanol). The samples were then separated by SDS-PAGE, transferred onto Immobilion-P^SQ^ Transfer Membranes (Merck Millipore, MA), blocked with NcmBlot blocking buffer (NCM Biotech, Suzhou, China) 20 min, washed three times with PBS containing 0.1% Tween 20 (Beyotime, Shanghai, China) (PBS-T), and incubated with primary antibodies overnight at 4°C. The membranes were washed three times with PBS-T and incubated with horseradish peroxidase (HRP)-labeled goat anti-rabbit IgG(H+L) for 1 h at room temperature. After washing three times with PBS-T, the TMB chromogen solution (Beyotime, Shanghai, China) was evenly added to the membranes and then exposed after standing for 2 min.

### Lentivirus production and transduction.

pLKO.1-PURO (carrying a puromycin antibiotic resistance gene) containing control, MFN1-shRNA, and MAVS-shRNA oligonucleotides were purchased from Tsingke Biotechnology (Beijing, China). Sequences of specific shRNAs used in this study were as follows: shMFN1-1, 5′-GAGATAAAGCCTATCTTAT (forward); shMFN1-2, 5′-CAAGATTACAGGAATTTCA (forward); shMAVS, 5′-CCAAAGTGCCTACTAGCAT (forward); and shMAVS, 5′-CACAGGGTCAGTTGTATCT (forward). Knockdown of MFN1 and MAVS in THP-1 cells was done by lentiviral transduction according to the manufacturer’s protocol and validated by Western blot analysis.

### Fluorescent confocal microscopy.

The cells were mixed with MitoTracker Red (1:10,000; ThermoFisher Scientific, Waltham, MA) and placed in the incubator at 37°C for 30 min in the dark according to the manufacturer’s recommended protocol. After several washes with PBS, the cells were incubated with 200 μL paraformaldehyde and 200 μL 0.5% Triton X-100 permeabilization solution for 15 min at room temperature. Then the cells were incubated with 200 μL blocking solution for 30 min at room temperature. After several washes with PBS, 200 μL MAVS primary antibody (1:200; Cell Signaling Technology, Boston, MA) was added to each tube overnight at 4°C. Then the cells were further incubated with anti-rabbit secondary antibody (1:1,000; Beyotime, Shanghai, China) and fluorescent secondary antibody (1:200; Proteintech, Wuhan, China) for 1.5 h at room temperature. Finally, the cells were dyed with 4′,6-diamidino-2-phenylindole (DAPI) (1:100; Beyotime, Shanghai, China) for 20 min, washed three times, then spun onto the slide, and sealed with an anti-fluorescence quencher. The images were obtained using a NikonA1 confocal microscope.

The cells were classified by measuring the length-to-width ratio of mitochondria. More than 50% of mitochondria in a cell showed that the length-to-width ratio over 20 was defined as the network type. More than 50% of mitochondria in one cell showed that a length-to-width ratio less than 20 was defined as the fragment type (32).

### Isolation of human peripheral blood monocyte cells.

Peripheral blood mononuclear cells (PBMCs) were isolated according to the methods described in the literature (33). Then the monocytes were separated by flow cytometry.

### Intracellular staining and flow cytometric analysis.

The cells were fixed with 4% paraformaldehyde in the proportion of 1 mL of 4% paraformaldehyde for every 2 × 10^6^ cells and fixed at room temperature for 10 to 15 min. Cell lotion was added, and the mixture was centrifuged at 1,000 rpm for 5 min. After washing several times with PBS, the cells were suspended with a film breaker (permeabilization buffer 10×; ThermoFisher Scientific, Waltham, MA) and added into a 1.5-mL EP tube (1 × 10^6^/tube, 100 μL/tube). The number of cells per tube of blank control, isotype control, and sample were 1 × 10^6^/tube. We then added 5 to 20 μL isotype antibody and target antibody to each tube, respectively (refer to the instruction for specific usage); mixed thoroughly; and then incubated at 4°C for 30 min or at room temperature for 30 min in winter. The reaction tube was shaken every 10 min during incubation, so that the cells and antibodies could fully react. After several washes with film breaker, the cells were further incubated with anti-rabbit secondary antibody (1:1,000; Beyotime, Shanghai, China) labeled with fluorescent (1:200; Proteintech, Wuhan, China) for 30 min at room temperature. Finally, the cells were washed with film breaker three times and then tested with flow cytometry.

### Ethical considerations.

This article does not contain any studies with human or animal subjects performed by any of the authors. All PBMCs were collected from healthy human donors.

### Data availability.

All the data and materials used in this report are included in the article.
