# Supplementary material for: Mitofusin 1-Mediated Redistribution of Mitochondrial Antiviral Signaling Protein Promotes Type 1 Interferon Response in Human Cytomegalovirus Infection
Source: Microbiol Spectr. 2023 Mar 20;11(2):e04615-22. doi: 10.1128/spectrum.04615-22 (PMC10100850; doi:10.1128/spectrum.04615-22)
Supplement: Supplemental file 1 — Fig. S1 to S3. Download spectrum.04615-22-s0001.pdf, PDF file, 0.5 MB [file spectrum.04615-22-s0001.pdf]

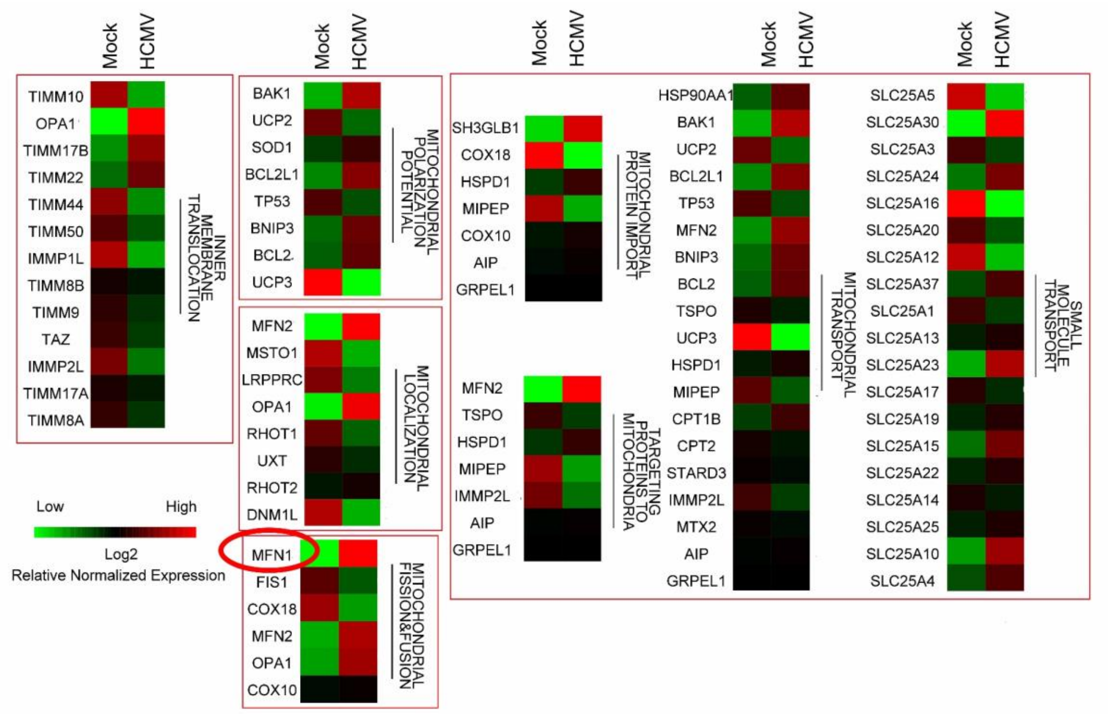

Supplementary Fig.1:

Heatmap analysis of mitochondrial related genes in control and THP-1 cells infected with HCMV (MOI=5) for 48h (data from GSE132048).

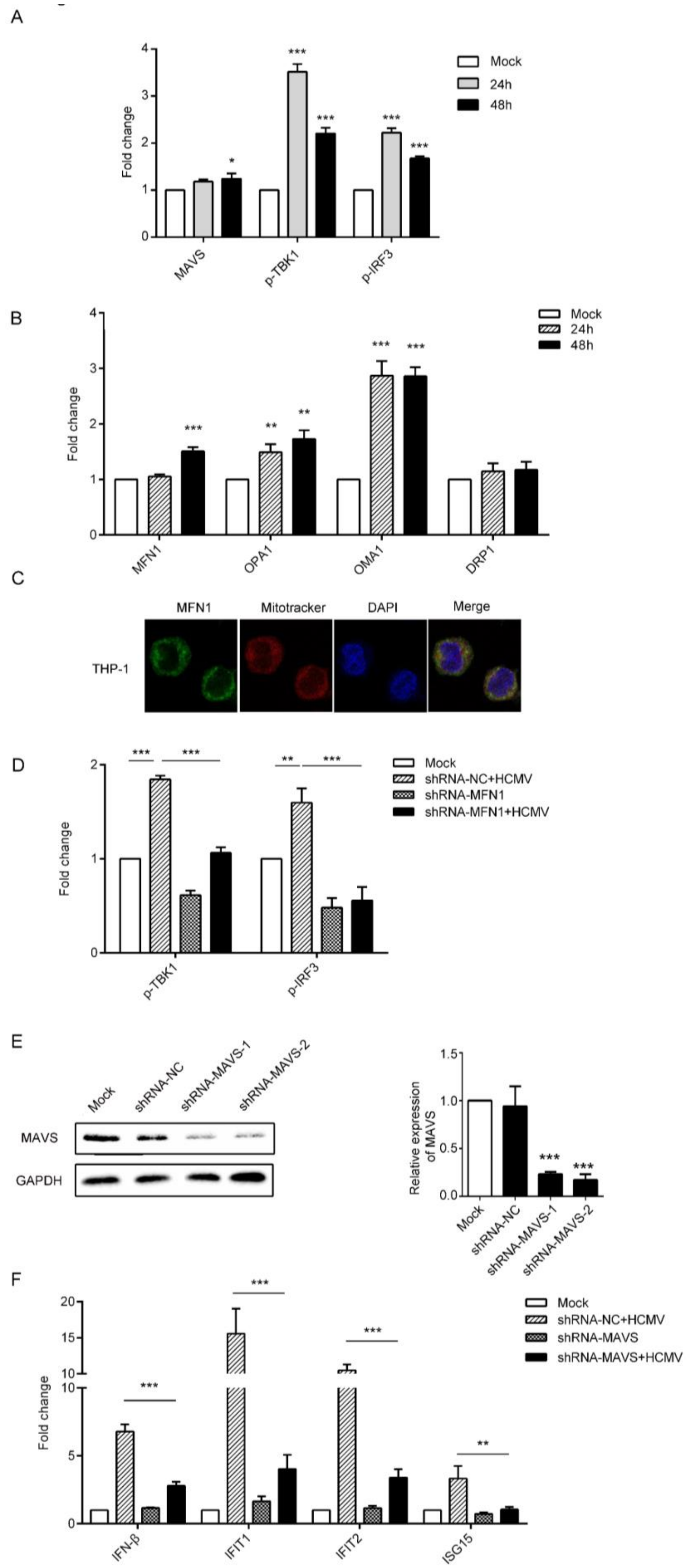
A
Fold change
Mock
24h
48h
MAVS
p-TBK1
p-IRF3
B
Fold change
Mock
24h
48h
MFN1
OPA1
OMA1
DRP1
C
MFN1
Mitotracker
DAPI
Merge
THP-1
D
Fold change
Mock
shRNA-NC+HCMV
shRNA-MFN1
shRNA-MFN1+HCMV
p-TBK1
p-IRF3
E
Mock
shRNA-NC
shRNA-MAVS-1
shRNA-MAVS-2
MAVS
GAPDH
Relative expression of MAVS
F
Fold change
Mock
shRNA-NC+HCMV
shRNA-MAVS
shRNA-MAVS+HCMV
IFN-β
IFIT1
IFIT2
ISG15

Supplementary Fig.2:

A. Quantitative analysis of the relative protein levels of MAVS, p-TBK1 and p-IRF3 in HCMV-infected THP-1 cells according to Fig.1B. **B.** Quantitative analysis of the relative protein levels of MFN1, OPA1, OMA1 and DRP1 in HCMV-infected THP-1 cells according to Fig.1D. **C.** THP-1 cells were stained with MitoTracker (Mitochondria) and anti-MFN1 antibody. Nuclei were visualized by staining with DAPI throughout this study. **D.** Quantitative analysis of the relative protein levels of p-TBK1 and p-IRF3 in control and MAVS-knockdown THP-1 cells according to Fig.2D. **E.** Western blot of MAVS in lysates from control and MAVS-knockdown THP-1 cells. **F.** The control shRNA treated and MAVs-knockdown THP-1 cells infected with HCMV (MOI=5) for 48 h, analyzed for *IFN-β*, *IFIT1*, *IFIT2* and *ISG15* mRNA expression relative to GAPDH mRNA by qPCR. Data are shown as the mean ± SEM of three independent experiments. NS (non-significant), $P>0.05$; $^*P<0.05$; $^{**}P<0.01$; $^{***}P<0.001$.

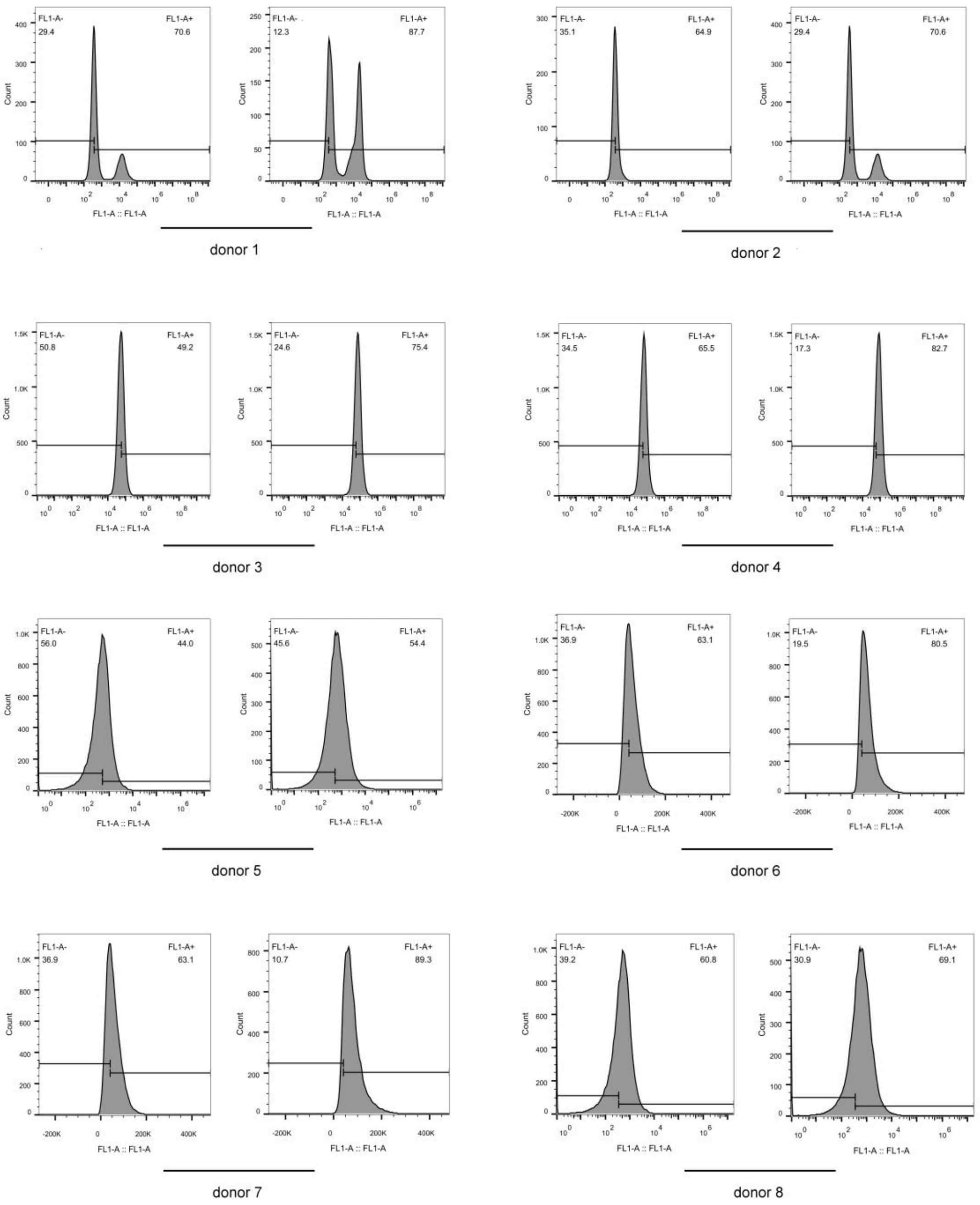

Supplementary Fig.3:

Detection of MFN1 expression in peripheral blood mononuclear cells of 8 donors infected with HCMV by flow cytometry
